# Arsenic in Soils Affected by Mining: Microscopic Studies vs. Sequential Chemical Extraction

**DOI:** 10.3390/ijerph17228426

**Published:** 2020-11-14

**Authors:** Jessica Álvarez-Quintana, Rodrigo Álvarez, Almudena Ordóñez

**Affiliations:** Mining Exploration and Exploitation Department, Escuela de Ingeniería de Minas, Energía y Materiales, University of Oviedo, 13th Independencia St, 33004 Oviedo, Spain; UO205184@uniovi.es (J.Á.-Q.); alvarezrodrigo@uniovi.es (R.Á.)

**Keywords:** contaminated soil, arsenic, mineralogy and texture of mining waste, sequential chemical extraction

## Abstract

Soil samples from three inactive mines, corresponding to different Arsenic-bearing mineralization types, were collected and studied. The aim was to determine the influence of mine wastes mineralogy/geochemistry and texture in As mobility and to compare results from sequential chemical extraction and microscopic techniques (optical and electron) at a grain scale. Arsenic in soils is found mainly associated to the residual fraction, indicating that mechanical As dispersion is mainly responsible for As soil pollution. The use of objective microscopic techniques (i.e., Scanning Electron Microscopy-Energy Dispersive Spectroscopy -SEM-EDS-, High Resolution Transmission Electron Microscopy -HR-TEM) has pointed out that the selected sequential extraction method overestimates the role of Mn amorphous oxy-hydroxides and organic matter in As retention while underestimating the mechanism of As adsorption onto clay particle surfaces.

## 1. Introduction

There is a large amount of scientific literature available that addresses the environmental aspects of mining waste. Concerning soil pollution, during the last decade of the 20th century, some generalist and multidisciplinary studies were published; these early investigations focused on the determination and spatial distribution of total concentrations of the elements of concern in each case. A significant advance in the approach, from a practical point of view, of polluted soil investigations, is constituted by the risk assessment methodologies proposed by the US Environmental Protection Agency at the end of the century. These protocols constitute a turning point from which more attention has been paid to the chemical forms in which the contaminants are present in the soil, highlighting that only the bioavailable fraction should be considered when calculating risk indexes.

Since then, the so-called “sequential extraction procedures” have been commonly considered by the scientific community as a useful tool to assess both the chemical speciation/mobility and the bioavailability of metals/metalloids from their association with certain solid phases. These techniques are basically chemical methods that employ different extractants (of growing strength in each step) that allow correlating each partial concentration with a mode of occurrence of the metal/metalloid in the soil (bound to Fe oxides, carbonates, organic matter). Some authors [1,2,3,4,5], among others, have pointed out that the correspondence between individual fractions and the real mineralogical bonding is not always reliable. An excellent critical summary of sequential extraction procedures can be consulted in the work of Zimmerman and Weindorf [5].

There are also some previous works [6,7,8,9,10,11,12] in which the results from sequential extraction are combined, generally in a wise way, with studies through conventional techniques in mineralogy: µ-X ray diffraction, scanning, and transmission electron microscopy, electron probe microanalysis, etc.; these investigations are in all cases carried out in relation to a specific case study (usually, historic metal mines).

On the other hand, the starting point to address the mobility of contaminants leads us to the original source, i.e., the mine wastes. Not many background investigations have focused their efforts on the importance and influence of mineral textures of mining wastes in relation to the degree of dispersion of pollutants in soils (see [13] for acid rock drainage prediction and some other references therein). In this work, a detailed examination of arsenical mine wastes and related soil pollution is presented. Objective results obtained by optical and electron microscopy techniques indicate that sequential extraction procedures should only be understood as an approach to reality. In addition to the above, this paper addresses the role of mine wastes mineral texture through the analysis of three clearly different arsenic ores.

## 2. Materials and Methods

### 2.1. Study Areas

**Figure 1:**
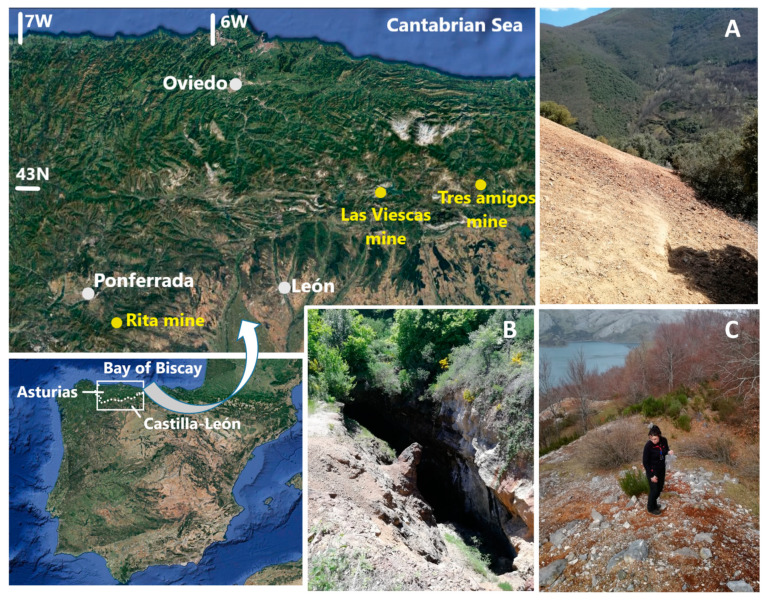
Location of main cities and mines cited in the text. (**A**) Spoil heap at Rita mine (Compludo, León); (**B**) Tres Amigos mine (Polentinos, Palencia); (**C**) spoil heap at Las Viescas mine (Riaño, León).

Three different mine sites, currently inactive, were selected for this study. All of them were included in the northern part of Castilla-León, a region that occupied most of the north-central area of the Iberian Peninsula (Figure 1). From a geological point of view, the first one was located in the León mountains, while the other 2 were within the Cantabrian Mountains, the westernmost mountain chain of Europe. Special care has been taken to select representative mineralization types, in which the presence of arsenic was unquestionable. A summary of the main characteristics of each mine site is presented below.

#### 2.1.1. Rita Mine

Rita mine was constituted by a series of old mining works located 250 m northeast from the village of Compludo (municipality of Ponferrada, Leon province, (X_UTM_ = 706,223; Y_UTM_ = 4,705,280, ETRS89, UTM zone 29). The mineralization appeared in the form of thin quartz veins that included arsenopyrite with small quantities of other sulfides, mainly pyrite. The economic interest of this mineralization was exclusively for As. These mineralized veins, not outcropping, were exploited in 4 levels (900–1040 m above sea level -a.s.l.) during the middle decades of the 20th century. Their general strike was N140E, and their average dip was about 80° (SW). Near each mining level, a spoil heap with low-grade ore, typically of several hundreds of m^3^ of volume, can be found (Figure 1A).

#### 2.1.2. Tres Amigos Mine

This mine site was found in the Carracedo mountain, above La Requejada water reservoir, about 2.5 km east of the small village of Polentinos, NW of Palencia province (X_UTM_ = 377,316; Y_UTM_ = 4,755,153, UTM zone 30). In this area, a high-temperature garnet-amphibolite skarn was developed in the contact of a series of diorite-like intrusive bodies and a level of carboniferous limestones locally known as “Brañosera Formation” [14]. The paragenetic mineral sequence was mainly constituted by chalcopyrite-arsenopyrite-magnetite-pyrite. In this case, unlike the other 2, the mineralization was exploited (until 1962) for Cu, and not for As. The mining works and associated spoil heaps (about 5000 m^3^) were distributed between 1100 and 1150 m a.s.l. (Figure 1B).

#### 2.1.3. Las Viescas Mine

Las Viescas mine (also known as “Bachende mine”) was located 2 km south of Riaño (NE of León province, X_UTM_ = 332,972; Y_UTM_ = 4,758,661, UTM zone 30). It exploited a low-temperature hydrothermal deposit hosted by Westphalian limestones (“Bachende Formation”) in which sulfide-rich irregular masses and veins were found. These mineralized bodies were exploited up to the 1950s for As. At present, 2 large spoil heaps were disposed on the slope (1150–1200 m a.s.l.), close to the fill level of the Riaño water reservoir, whose capacity was 664 Mm^3^. Mine wastes (rock fragments) were especially rich in arsenopyrite and pyrite in this case. Additionally, the presence of marcasite, stibnite, Sb-ochres, and realgar had also been cited at this site [15].

### 2.2. Sampling and Analysis

**Figure 2:**
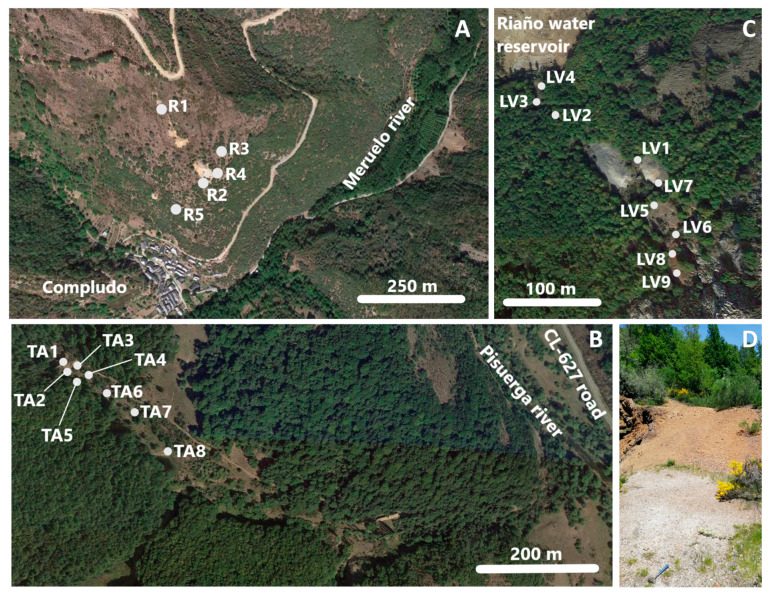
Location of sampling points. (**A**) Rita mine (samples R1 to R5); (**B**) Tres Amigos mine (samples TA1 to TA8); (**C**) Las Viescas mine (samples LV1 to LV9); (**D**) location of sampling point TA1 in a spoil heap at Tres Amigos mine (pointed by the hammer).

In the 3 mine sites described in the previous section, some soil samples were taken for analysis: 5 at Rita mine, 8 at Tres Amigos mine, and 9 at Las Viescas mine (Figure 2, the number of samples depends on particular site characteristics, mainly the size). Sampling points were selected as representative, a priori, of the more affected areas: Zones adjoining to old mining works and/or located downstream from the main spoil heaps. The objective of the sampling campaign was not to precisely quantify the pollutant contents and their spatial distribution, but to substantiate the study from samples with high As (and other metals) contents, thus that it would be possible to determine As mode of occurrence in soil particles.

Soil samples were taken by means of a steel auger-type sampler, recovering 20 cm of the sample from the surface. Once in the laboratory, soil samples were prepared for analysis following the conventional protocols of drying, removal of vegetable matter, soil particles disaggregation, and sieving.

Jointly with representative samples of mine wastes from each mine site, soil samples were used to prepare polished sections for microscopic studies. The fraction below 125 µm of each soil sample was analyzed by energy-dispersion X-ray fluorescence (EDXRF) by a portable Niton XL3t analyzer (Thermo Fisher Scientific, Waltham, MA, USA) that incorporated a silicon drift detector. Polished sections were studied and analyzed by optical and electronic microscopy using a Leica DMLP petrographic microscope (Leica Microsystems, Wetzlar, Germany) and a JEOL JSM-5600 Scanning Electron Microscope (SEM, JEOL Ltd., Tokyo, Japan) the latter endowed with an Oxford Inca Energy 200 EDS module (Oxford Instruments, Abingdon, UK). Finally, 20 g of each sample were grounded to a size below 63 µm and sent to Bureau Veritas Mineral Labs at Vancouver to carry out the sequential extraction analyses (ICP-MS), following the procedure detailed in Hall et al. (1998) [16]. Selected sub-samples were dispersed in ethylene glycol and studied by a JEOL JEM-2100 HRTEM (JEOL Ltd., Tokyo, Japan) at the Scientific and Technical Services Labs of the University of Oviedo.

## 3. Results and Discussion

### 3.1. Mineralogy and Texture of Mine Wastes

Mineralogical and textural analyses of mine wastes were carried out by examining polished sections under reflected polarized light using a conventional petrographic microscope (Leica Microsystems, Wetzlar, Germany).

#### 3.1.1. Rita Mine

**Figure 3:**
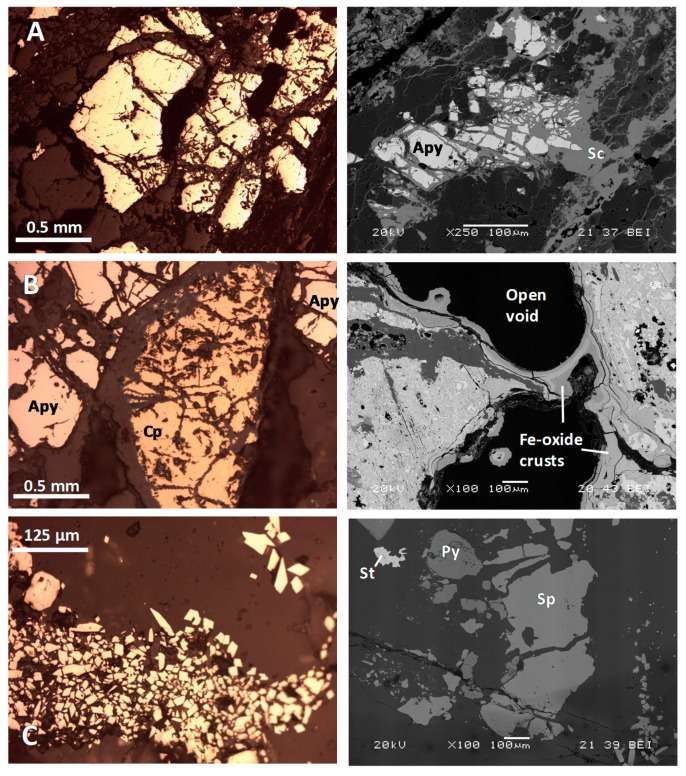
Mine wastes microtexture. Each line combines pairs of images captured by optical microscopy (left column) with SEM backscattered-electron images (right column). (**A**) Rita mine: Individual arsenopyrite crystals (left image) and scorodite (Sc) neoformation (from arsenopyrite (Apy) weathering (right image). (**B**) Tres Amigos mine: Primary arsenopyrite (Apy) and chalcopyrite (Cp, left), and iron oxide crusts partially filling open spaces (right). (**C**) Las Viescas mine: Idiomorphic arsenopyrite crystals in a calcite gangue (left) and disperse sphalerite (Sp)-pyrite (Py)-stibnite (St) (right).

Mine wastes stored at Rita mine spoil heaps were particles (0.5–20 cm) of low-grade ore accompanied by rock fragments of the enclosing schists. Low-grade ore materials were angular fragments of milky, large quartz crystals, in which some arsenopyrite crystals were frequently found. These ore showed the typical features of the well-known greisen type mineralization: Thin quartz veins within a pelitic sequence, with muscovite crystals in their contact and poor mineralization in which arsenopyrite was practically the only or almost the sole sulfide. Arsenopyrite grain size varied from 0.1 to more than 1 mm of diameter. This sulfide generally appeared as sub-idiomorphic primary crystals affected by locally intense transgranular fractures and with different stages of weathering to form scorodite (Figure 3A). Scorodite growth was recognizable in the contour of transgranular fractures affecting arsenopyrite crystals and, to a lesser extent, in the external borders of the arsenopyrite crystals. Scorodite was much better developed when the mineralization affects the enclosing schist (Figure 3A), being almost negligible in quartz vein samples. Other sulfides that could be identified, although in very scarce quantities, were pyrite, Cd-rich sphalerite, and galena.

#### 3.1.2. Tres Amigos Mine

Mine waste samples taken at Tres Amigos mine spoil heaps showed relevant Cu-As mineralization, with chalcopyrite and arsenopyrite as primary sulfides, accompanied by pyrite and hydrothermal quartz (as well as other oxides) in variable quantities. Both the chalcopyrite and the arsenopyrite appeared as millimetric crystals, intensely fractured (Figure 3B). Fracture planes were preferred zones for the development of supergene mineral transformations that included the neoformation of scorodite from arsenopyrite and the formation of thin (up to 30 µm thick) covellite coatings over chalcopyrite. A significant amount of the total mineralized rock fragments of the spoil heaps at the Tres Amigos mine showed an advanced state of supergene alteration revealed by the abundance of crustiform-banded precipitates of iron oxides (probably poorly crystalline, Figure 3B). These iron oxide crusts contain high quantities of As (1–7% dry weight -wt).

#### 3.1.3. Las Viescas Mine

The study of both the mine’s geological context and the mine wastes showed unmistakable signs that the ore exploited at Las Viescas mine was a hydrothermal-type deposit (magmatic affinity). The host rock (limestone) presented big-sized calcite crystals, with evident recrystallization. This was the mineralization richest in As of all those studied in this work: It presented a massive character, with an arsenopyrite (-pyrite) dissemination over a large rock volume. Individual arsenopyrite crystal size was very small (about 40 µm as an average value, Figure 3C), but arsenopyrite crystal aggregates can reach millimetric sizes. SEM-EDS analyses have pointed out that the pyrite, frequent in this deposit, is also As-rich. Together with pyrite and arsenopyrite, stibnite and Cd-rich sphalerite are also present in the mineral paragenesis (Figure 3C). All the sulfides showed a sub-idiomorphic shape, excepting the arsenopyrite, which was always idiomorphic.

### 3.2. Soils Geochemistry

As described in Section 2.2, soil samples were analyzed to determine metal and metalloid contents by EDXRF at the University of Oviedo. X-ray (40 kV) excitation time for each analysis was 90 s, and the calibration mode selected for the equipment was “soil”. Results expressed in mg/kg are shown in Table 1.

As expected by the sampling points selection, As contents in soil samples were very high. An immediate conclusion was that As mobility was higher in the case of the Las Viescas mine. This fact can be easily explained by the mine wastes texture described in Section 3.1: The host rock (limestone) was soluble, and arsenopyrite was dispersed in the form of small euhedral crystals of 40 µm of diameter (Figure 3C), with a high specific surface area, thus it was much more exposed to chemical weathering than arsenopyrite from the Rita and Tres Amigos mines. Soils from the Rita mine exhibited the lowest As contents due to the role that crystalline quartz plays in relation to arsenopyrite weathering: It acts as an efficient protecting agent. Wastes from the Tres Amigos mine would be in an intermediate context: Gangue combine soluble (hosting limestone), and insoluble (skarn silicates) components and the arsenopyrite appeared in large size crystals, although they were fractured. Results for Fe followed the same trend, but the analysis, in this case, was not as simple as for As, as Fe origin was not only associated with dispersion from arsenopyrite. Iron contents in soil from the Rita mine were within usual ranges for a common soil (5.4%) [18], while in the other two mine sites, Fe soil concentrations were clearly influenced by the presence of Fe-oxide particles (discussed later) whose origin was presumably related to mining waste. The Castilla y León region (Figure 1) has not yet developed regional reference concentrations for polluted soil. Notwithstanding, the reference values of the neighboring Asturias region [17], which was placed at the Northern border of Castilla y León (Figure 1), with similar geologic characteristics (both areas were within the so-called “Cantabrian Zone” [19]), were considered.

The above-mentioned reference values, considering the less restrictive use (“industrial”), were exceeded for Sb in the Tres Amigos and Las Viescas mines, and also for Hg in the latter. On the other hand, many samples exceeded the Cu threshold value (4000 mg/kg, see Table 1). Cu content was much higher in the soil of Tres Amigos mine, which can be explained by the mineralogy and texture of the mining waste sampled there. In this case, the leachability of As from arsenopyrite was greater than that of Cu from chalcopyrite, since the primary minerals of both elements appeared in similar proportions and grain sizes, the presence of As being much more evident in the surrounding soils.

### 3.3. Sequential Extraction for As

Sequential extraction (or “sequential fractionation”) analyses have been performed in order to know in detail the mode of occurrence of As and other metals in soil samples. Since the first sequential extraction protocols, developed by Tessier and collaborators in the late 1970s [20], innumerable variants appeared that tried to improve some of the weak points of these procedures, mainly the selectivity of the extracting agents. In this case, the Hall method [16] has been selected against others equally recognized [21,22,23,24,25] because, in addition to the As fractions associated with clay phases, organic matter, and carbonates, it allows to establish certain differences between the fractions associated with Mn and Fe oxides. The first fraction (F1) was soluble in demineralized water; to obtain the second one (F2), ammonium acetate 1M was used, and it was interpreted as the fraction of As adsorbed in clays or co-precipitated in the form of carbonates. The third fraction (F3) was obtained by digestion with sodium pyrophosphate, and it was assigned as associated with organic matter. The fraction F4 was associated with amorphous Mn hydroxides, and the remaining fraction (F5) was linked to amorphous and crystalline Fe oxyhydroxides and crystalline Mn oxyhydroxides; these two last fractions were obtained by means of digestion (extraction) with hydroxylamine 0.1 and 0.25 M, respectively.

The samples from the three mines were submitted to this sequential extraction. Recovered extracts were analyzed by ICP-MS, and the results for As are shown in Table 2.

If the total As results (third column of Table 1) are compared with those of Table 2, some deductions can be made. First of all, the difference between the sum of the fractions in Table 2 and the total content of As could be assimilated to the so-called “residual” As; i.e., the As that formed its own mineral species (see [26] for an exhaustive collection of As species in soils), or the As that was found as an impurity in the crystalline lattice of other non-arsenical mineral phases. In most samples, the residual As would represent more than half of the As content. As an average, 59% of the As in the soil samples from Rita mine is residual. Analogously, this value would be 50% and 84% in the Tres Amigos and Las Viescas mines, respectively.

This indicated that most of the As in the soil was due to mineral particles from mining wastes, thus, the predominant dispersion was mechanical instead of chemical. In fact, the slope of the terrain followed the same trend as the residual As concentration: Las Viescas > Rita > Tres Amigos. The amounts of As in free phase or adsorbed in clays were, in relative terms, very small (always below 2%), despite the strongly clayey character of some of the samples (for example, LV8 and LV9). The percentages of As ideally associated with organic matter varied in very broad limits, resulting in lower percentages for the samples from Las Viescas mine, in average terms. None of the samples seemed to contain significant amounts of organic matter; in contrast and according to the sequential extraction results (Table 2, column F3), organic matter should play an important role in the capture of As in some specific samples (TA3, TA5, TA8, among others, see next section for a detailed explanation). The values and trends obtained for the fraction of As associated with amorphous or poorly crystalline hydroxides of Mn are similar, in general, to those determined for organic matter. This relative similarity is remarkable, taking into account that these are different mechanisms that compete for the As. There was not a good correspondence between the total Mn present in each sample and the As released in fraction F4 (Pearson correlation coefficient = 0.38). Additionally, in some samples (e.g., LV3-LV6, LV8, or LV9), total Mn (adsorbent, Table 1) values were lower than those for As (adsorbate) in F3; therefore, single adsorption onto Mn hydroxides mechanism was not possible. It would be interesting to determine the form of the Mn found in the studied soils. The fraction that contains the highest amount of As was the last one (F5), that corresponds to the As associated to Fe oxides/hydroxides. The correlation between total Fe in each sample and the As contained in this fraction (F5) showed a good correlation (r = 0.78, *n* = 22, *p*-value < 0.01). It should be taken into account that the amount of total Fe in the soil samples was much higher than that of Mn; assuming that both transition metals were mainly present in the samples in the form of oxides and hydroxides, Mn oxy-hydroxides appear to be more effective As collectors than Fe oxy-hydroxides. Hall [16] indicates some chemical reasons that support this fact. Finally, it should be noted that the samples richest in As have a very high residual fraction since otherwise, it would be difficult to explain contents higher than 10% of elemental As in a soil.

### 3.4. SEM and HRTEM Studies

In order to determine the goodness of the interpretations of the sequential extraction techniques, electron (scanning and transmission) microscopy studies were carried out on some representative samples. SEM and TEM equipment (see Section 2.2) works al 30 kV and 200 kV, respectively. The selected mode of image for SEM was backscattered electron detection. The resolution of EDX modules of analysis was 125 eV in both cases. The microanalysis by means of dispersive energy has been employed in order to confirm As presence or absence in individual solid particles, almost without size limitation in the HRTEM. The following comments are ordered according to the quantitative importance of the fractions described in the previous section.

**Figure 4:**
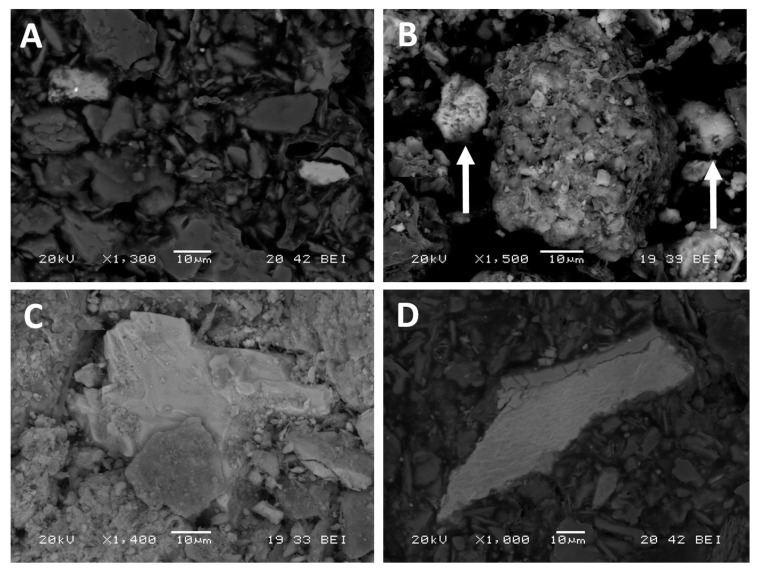
SEM images of selected grains (backscattered electron mode). (**A**) Soil mineral particles from Rita mine site: Those with higher atomic weight show higher brightness (scorodite with a residue of arsenopyrite on the left and a Fe-oxide on the right). (**B**) Two small particles of Fe-oxides from Las Viescas mine (marked with arrows), whose As contents are 2.74% and 8.76%, respectively. (**C**) Fe-oxide subhedral grain from Tres Amigos mine with a content of 2.80% As. (**D**) Mn-oxide grain from Rita mine in which no As was detected.

Firstly, the predominance of the residual phase that was previously indicated is evident in the observation of the soil samples with SEM using backscattered electrons: As-rich particles are very frequent; they are variable in size, almost always angular and generally homogeneous, constituted by sulfides, oxides and intermediate phases of As-Fe (Figure 4A).

EDX microanalyses with SEM indicate that Fe oxides/hydroxides present in soil samples contain As in proportions up to 11%, with most values around 2–3% (Figure 4B,C). Double Fe-Mn oxides are also frequent. As a simplification, if we consider the total Fe of the soil samples (Table 1) and As associated with oxides/hydroxides of Fe in each sample (Table 2, column F5), the contents of the metalloid in these phases would be around 1.7% (0.1–6.3%) on average. Therefore, we can accept the validity of the procedure used in the sequential extraction in relation to this fraction. Previous studies [27] revealed that the As associated with amorphous Fe oxy-hydroxides is, in general, much higher than that associated with the same compounds when these have a crystalline structure.

An analogous procedure could be applied to the As fraction associated with poorly crystalline Mn oxides/hydroxides. The ratio between the As obtained in this fraction (F4, Table 2) and the total Mn in the soil samples (Table 1) is 38.4%. Even assuming that all Mn is in this form, EDS microanalyses do not provide, in any case, such high As/Mn ratio; the content of As in oxides/hydroxides of Mn usually does not exceed 2%, being zero in many cases (e.g., Figure 4D). Therefore, for this fraction (F4), the objectivity of the SEM-EDS leads to question the interpretation of the sequential chemical extraction results.

The organic matter values in the soil samples were generally low. It is difficult to find particles of organic compounds in the soil samples observed by SEM. This scarcity, together with the absence of As spectral lines in the microanalyses (which means an As content <0.5%) leads to think, again, that the amount of As that sequential chemical extraction associates with organic matter are greatly overestimated.

**Figure 5:**
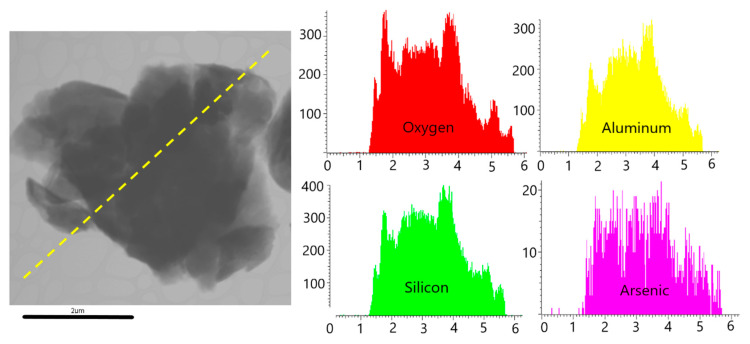
HR-TEM image of a clayey particle in sample R2. The yellow line indicated the section of the analysis. Graphs on the right show the distribution of O, Al, Si, and As. Note the difference between the vertical scales of the elements’ graphs.

The role of clays (mostly kaolinite, as it was determined by X-ray diffraction) as adsorbents seems to be moderate, considering the results obtained for As in the second step of the sequential extraction (F2). Isolated clay particles were very difficult to study separated from the rest of the soil materials in the SEM preparations. However, when they were observed and microanalyzed with TEM, As contents between 0.5% and 1% were found, especially in the clay particles with certain Fe content. As an example, Figure 5 shows how As is distributed more or less homogeneously over the entire surface of the sheet that constitutes each individual particle. Thus, contrary to what happens with organic matter, the employed method of sequential extraction underestimates the As content associated with clay minerals.

## 4. Conclusions

Three As-polluted mine sites have been studied combining chemical and mineralogical/geochemical methods: Sequential chemical extraction vs. optical and electron microscopy. It has been verified that As-pollution intensity in soils is strongly influenced by mine wastes (original As source), geochemistry, and microtexture; on the other hand, As spatial distribution mainly depends on specific site topography. Among the factors that govern and promote As chemical dispersion, host rock nature (limestones/marbles or siliceous rocks) and arsenopyrite grain size should be considered as dominant.

Results from sequential chemical extraction for As point out that the majority of this metalloid in soils occurs in the so-called “residual fraction”. That is, most of the As present in soils at abandoned mine sites is found in grains of specific As minerals that come from the wastes and are later integrated within the soil mineral fraction. Then, mechanical dispersion is quantitatively more important than chemical dispersion.

Results from the rest of the chemical fractions obtained by sequential extraction were compared with observations at grain scale by means of SEM and TEM. The highest percentage of As retention in soils is found in fraction F5 (Fe oxides and hydroxides), an aspect equally notifiable by means of SEM. Arsenic ideally associated with Mn oxides and hydroxides (fraction F4) and also to organic matter (fraction F3) accounts for 10.6% and 7.3% of total As, respectively, according to sequential chemical extraction results. This interpretation does not match well with the fact that As is almost inexistent (or undetectable) when SEM microanalyses are carried out over Mn oxides or organic grains surfaces. Concerning As associated with clay minerals (fraction F2), it is almost negligible according to the sequential chemical extraction (0.7% of total As), whereas TEM analyses have revealed a significant role of clays in As fixation since these particles are abundant in the samples (SEM observations) and they contain around 1% of As.

Sequential chemical extraction protocols constitute a useful tool in soil pollution characterization, but the mineralogical interpretation of the obtained fractions should only be understood as an approach to reality. In this paper, this fact has been highlighted in the fractions of As associated to organic matter and Mn oxy-hydroxides (both overestimated) and also in the fraction of As associated to clay minerals (underestimated).

As future perspectives, it is important to point out that some authors who have dealt with the mineralogical speciation of As in soil samples make use of other techniques, such as X-ray diffraction (which only provides the majority constituents), X-ray adsorption spectroscopy (XAS), or X-ray photoelectron spectroscopy (XPS, none provide mineralogical data), and the synchrotron-based micro-focused μ-XRD [6,28,29,30,31,32,33]. A significant advance could come from the preparation of suitable samples to be studied by electron probe micro-analysis (EPMA); the soil constituents are too soft to be studied by this technique, which provides a higher analytical resolution than that of electron microscopy. On the other hand, it would be desirable to develop a procedure that would allow the As (or other elements) in the sample to be globally quantified by combining electron microscopy images and microanalysis. It would also be of interest to carry out mineralogical/geochemical studies on the solid waste that is recovered after the different stages of sequential extraction.

## Figures and Tables

**Table 1:** Selected metal(loid)s content in soil samples (in mg/kg; ND: Not detected). Std deviation is indicated in brackets below each value. Reference value according to the less restrictive use of soil (industrial) [17].

Mine	Sample	As	Cu	Fe	Hg	Mn	S	Sb
Rita	R1	615.1 (±12.5)	32.8 (±10.3)	27,942.4 (±219.6)	ND (<7.1)	1178.8 (±64.4)	ND (<301.5)	ND (<20.4)
	R2	9026.4 (±45.5)	67.4 (±11.6)	41,827.5 (±268.4)	11.1 (±6.7)	1743.4 (±76.7)	1747.3 (±335.8)	ND (<16.7)
	R3	2015.5 (±22.2)	50.7 (±11.1)	31,665.8 (±238.1)	ND (<7.7)	1414 (±70.7)	419.9 (±206.3)	ND (<19.3)
	R4	721.8 (±13.1)	38.3 (±9.7)	29,435.8 (±215.1)	ND (<6.7)	3495.8 (±97.1)	700.5 (±239.9)	ND (<17.7)
	R5	12,840.2 (±68.2)	41.5 (±14.9)	138,906.9 (±607.8)	ND (<14.1)	5700.8 (±168.1)	965.4 (±392.4)	ND (<25.3)
	Mean	5043.8	46.1	53,955.7	11.1	2706.6	958.3	
Tres Amigos	TA1	20,173.6 (±96.9)	6571.2 (±99.6)	189,922.1 (±826.4)	ND (<19.0)	4063.4 (±175.2)	1484.7 (±483.7)	532.2 (±27.4)
	TA2	51,330.8 (±207.8)	3114.7 (±95.1))	489,491.5 (±1779.4)	ND (<35.4)	1526.8 (±215.5)	3436.4 (±652.3)	958.6 (±37.9)
	TA3	1064.8 (±15.3)	863.8 (±25.5)	30,370.8 (±223.1)	ND (<6.9)	349.7 (±42.5)	ND (<302.5)	ND (<20.3)
	TA4	4513.6 (±35.2)	2644.8 (±48.8)	60,453.5 (±356.7)	ND (<9.6)	863.2 (±68.2)	ND (<397.1)	105.3 (±17.2)
	TA5	1927.6 (±20.9)	509 (±20.9)	41,561.2 (±266.9)	ND (<7.5)	1158.4 (±65.3)	553.5 (±252.7)	ND (<20.3)
	TA6	4136.8 (±32.1)	3631.8 (±54.0)	50,032.9 (±309.5)	ND (<9.1)	1655.7 (±80.4)	913.5 (±308.1)	118.4 (±15.8)
	TA7	12,249.1 (±69.2)	6843.6 (±92.8)	150,006.5 (±671.9)	ND (<15.7)	2326.9 (±127.4)	786.3 (±374.7)	335.3 (±23.2)
	TA8	1774.8 (±21.3)	1597.2 (±36.6)	48,058.7 (±303.7)	ND (<8.1)	952 (±65.4)	ND (<356.7)	ND (<21.9)
	Mean	12,146.4	3222	132,487.2		1612	1434.9	410
Las Viescasss	LV1	46,810.1 (±164.2)	297.6 (±32.0)	143,313.3 (±797.4)	49.7 (±20.0)	508.2 (±105.9)	3853.1 (±604.8)	312.5 (±27.4)
	LV2	972.1 (±15.1)	31.1 (±10.4)	36,110.6 (±252.0)	ND (<7.1)	1088.4 (±63.9)	ND (<383.9)	ND (<21.5)
	LV3	26,902.6 (±99.9)	109.3 (±18.3)	91,556.6 (±511.6)	44.6 (±12.8)	1064 (±90.6)	3291.7 (±466.0)	ND (<23.7)
	LV4	65,978.7 (±215.3)	235.8 (±34.6)	203,996.3 (±1050.5)	74.6 (± 25.1)	1039 (±146.7)	7045.2 (±690.4)	176.8 (±24.9)
	LV5	140,149.9 (±425.4)	526.3 (±65.2)	488,617.3 (±2205.1)	159.8 (± 48.2)	1759 (±279.6)	5112.6 (±730.7)	328 (±33.3)
	LV6	131,303.1 (±429.4)	663.4 (±72.2)	701,787.4 (±2752.7)	148 (±48.7)	2117.9 (±334.2)	1999.7 (±650.4)	311 (±34.1)
	LV7	47,711.4 (±168.4)	283.8 (±32.3)	191,690.7 (±936.6)	31.2 (±20.1)	1575.5 (±145.7)	3116.2 (±571.9)	224 (±25.3)
	LV8	155,169.7 (±489.6)	597 (±75.3)	706,385.8 (±2897.4)	206.6 (±55.8)	1772.8 (±343.1)	1773.3 (±619.5)	332 (±34.9)
	LV9	180,545.1 (±530.4)	523.7 (±74.9)	571,594.1 (±2620.6)	171.9 (±58.6)	1197 (±307.2)	6862.1 (±806.5)	392.9 (±36.5)
	Mean	88,393.6	363.1	348,339.1	110.8	1346.9	4131.7	296.9
Reference value [17]		200	4000	ND	100	9635	ND	295

**Table 2:** As contents in the different fractions for each sample.

Fraction	1		2		3		4		5	
Sample	Conc. (mg·kg^−1^)	% of Total As	Conc. (mg·kg^−1^)	% of Total As	Conc. (mg·kg^−1^)	% of Total As	Conc. (mg·kg^−1^)	% of Total As	Conc. (mg·kg^−1^)	% of Total As
R1	2.3	0.37	3.6	0.58	71.30	11.6	74.6	12.1	56.6	9.21
R2	74.6	0.83	84.9	0.94	788.5	8.74	635	7.04	2648	29.3
R3	34.0	1.68	38.4	1.90	452.2	22.4	266	13.2	253.9	12.6
R4	8.90	1.23	7.60	1.05	84.60	11.7	61.0	8.46	102.3	14.2
R5	48.8	0.38	55.6	0.43	754.0	5.87	964	7.51	2607	20.3
TA1	18.1	0.09	91.8	0.46	1023	5.07	1640	8.13	3195	15.8
TA2	4.80	0.01	73.5	0.14	3626	7.06	1096	2.14	4798	9.35
TA3	4.50	0.42	15.1	1.42	331.3	31.1	189.7	17.8	300.0	28.2
TA4	18.2	0.40	41.3	0.92	532.1	11.8	730.2	16.2	898.4	19.9
TA5	4.90	0.25	12.4	0.64	655.4	34.0	208.6	10.8	414.5	21.5
TA6	14.4	0.35	49.7	1.20	465.5	11.2	405.6	9.81	987.9	23.9
TA7	40.3	0.33	80.6	0.66	858.1	7.01	1523	12.4	1726	14.1
TA8	7.80	0.44	7.00	0.40	391.5	22.1	267.6	15.1	601.2	33.9
LV1	92.3	0.20	115	0.25	1227	2.62	205.9	0.44	3134	6.70
LV2	3.30	0.34	11.8	1.21	105.4	10.8	81.90	8.42	135.8	14.0
LV3	30.7	0.11	209	0.78	3210	11.9	1809	6.73	5782	21.5
LV4	119	0.18	415	0.63	2076	3.15	627.8	0.95	6168	9.35
LV5	144	0.10	545	0.39	4385	3.13	603.6	0.43	7270	5.19
LV6	162	0.12	511	0.39	4446	3.39	719.3	0.55	5241	3.99
LV7	75.9	0.16	142	0.30	1571	3.29	1005	2.11	4280	8.97
LV8	169	0.11	604	0.39	4261	2.75	1124	0.72	6272	4.04
LV9	155	0.09	558	0.31	4298	2.38	544.4	0.30	5866	3.25
